# Was It a Vision

**Published:** 1889-08

**Authors:** Amarala Martin

**Affiliations:** Chicago, Ill.


### WAS IT A VISION ?

We were having a new home built and my husband went to it every day to see what progress was being made. One day, as he stood in the front room upstairs his attention was attracted to the street. Looking out the window he saw a funeral procession passing from the door and out through the gate. The casket was small, white, and covered with flowers. He recognized friends and neighbors in the crowd, and through some indefinable impression, he understood that the corpse was that of his *son*. His son, though he had no son at the time. Surprised and startled at the thought, he saw the procession vanish, and he felt unable to account for the experience.

Within a few months a little son was born to us, and before three years it was carried out the gate in a flower-covered, white casket and followed by the same friends my husband had seen that noontime long before. What explanation can be given of this circumstance?

Chicago, Ill. AMARALA MARTIN.

